# Fungal Biofilms 2020

**DOI:** 10.3390/jof7080603

**Published:** 2021-07-26

**Authors:** Célia F. Rodrigues, Jesus A. Romo

**Affiliations:** 1 LEPABE—Laboratory for Process Engineering, Environment, Biotechnology and Energy, Faculty of Engineering, University of Porto, Rua Dr. Roberto Frias, 4200-465 Porto, Portugal; 2 Department of Molecular Biology and Microbiology, Tufts University School of Medicine, Boston, MA 02111, USA

Fungal infections are an important and increasing global threat, carrying not only high morbidity and mortality rates, but also extraordinary healthcare costs. Without an effective response, it is predicted that 10 million people will die per year because of multidrug-resistant pathogens. A high percentage of the mortalities caused by fungi are known to have a biofilm etiology [1,2,3,4]. In fact, biofilms are the predominant mode of fungal growth. They have several ecologic benefits, for example higher nutrient availability, metabolic cooperation, protection from the environmental stresses, and acquisition of new and advantageous features. Besides, single-species and mixed-species biofilms are particularly problematic to eradicate, being, thus, the foundation of chronic infections, particularly if medical devices are existent [5].

A total of ten papers were published in this Special Issue including three reviews and six original articles. These cover a wide range of topics with original research on polymicrobial biofilms of fungi and bacteria, small molecule screening, characterization of the impact of current antifungals on biofilms of non-*albicans* species, characterization of non-*albicans* species biofilm matrix, and biofilms of *Aspergillus fumigatus.* Additionally, review articles cover the antifungal effect of *quorum sensing* molecules on *Candida* biofilms, sexual biofilms of *Candida albicans*, and a compilation of plant derived compounds and their activities against biofilms formed by *Candida* species.

The reports describe original research in the area of antimicrobials and include work involving individual and combinatorial efficacy of compounds with specific activity against fungi, bacteria, or both within polymicrobial biofilms [6], a screen of a small molecule library alone or in combination with current antifungals in search of compounds with anti-biofilm and pre-formed biofilm activities [7], characterization of the effect of echinocandins against planktonic and biofilm lifestyles of clinical isolates from the *Candida haemulonii* complex [8], and the use of a membranotropic peptide to disrupt polymicrobial biofilms of *Candida albicans* and *Klebsiella pneumoniae* [9]. Additional reports phenotypically characterized colonies from *Candida parapsilosis* clinical isolates as a way to predict their biofilm formation capabilities [10], conducted analyses of the biofilm matrix composition from the *Candida haemulonii* species complex [11], and investigated the virulence and biofilm capabilities of an *Aspergillus fumigatus* environmental isolate with interest in the role of this isolate in the textile industry [12].

The reviews in this Special Issue covered recent developments in the area of *Candida albicans* sexual biofilms specifically focusing on how they are formed, their physical characteristics, and their role in *Candida* biology [13], the properties of the quorum sensing molecules farnesol and tyrosol secreted by *Candida* and their effect as anti-biofilm agents [14], and an extensive compilation of plant derived compounds with activities against biofilms of distinct *Candida* species [15]. Overall, this Special Issue is a great resource highlighting novel work on fungal biofilms.
